# Novel terahertz optical switch based on PIT phenomenon and Lorentz theory

**DOI:** 10.1016/j.isci.2024.111301

**Published:** 2024-11-01

**Authors:** Jun Zhu, Xiner Chen, Liuli Qin

**Affiliations:** 1 Guangxi Key Laboratory of Brain-inspired Computing and Intelligent Chips, School of Electronic and Information Engineering, Guangxi Normal University, Guilin 541004, China; 2 School of Electronic and Information Engineering/School of Integrated Circuits, Guangxi Normal University, Guilin 541004, China; 3 Key Laboratory of Advanced Electrode Materials for Novel Solar Cells for Petroleum and Chemical Industry of China, School of Chemistry and Life Sciences, Suzhou University of Science and Technology, Suzhou City, Jiangsu Province 215009, P.R. China; 4 School of Mathematics and Statistics, Guangxi Normal University, Guilin 541004, China

**Keywords:** Physics, Optics, Electromagnetics

## Abstract

We propose and demonstrate a structure consisting of graphene rings and square rings that enables broadband and tunable plasmon-induced transparency (PIT) effects. Through coupled Lorentz model analysis, we attribute the transmission window at 2.1 THz to the interference between the equipartitioned exciton resonance of the graphene ring pairs and the inductive-capacitive resonance of the graphene square ring pairs. We also investigate the effect of the variation of the rotation angle of the internal graphene square ring pair on the transmission characteristics. The structure not only achieves a maximum modulation depth (MDA) of 91%, insertion loss (IL) and extinction ratio (ER) of 0.3 dB and 10.94 dB, respectively, but also achieves a maximum detection sensitivity of 0.96 THz/refractive index unit (RIU). In contrast, this study achieves more than 90% modulation amplitude in the range of 0.3 THz with a simple design structure, providing new insights for research and applications in related fields.

## Highlights

• Graphene-based metamaterial achieves wideband tunable PIT effect
• Performance: MDA of 91%, ER up to 10.94 dB, IL value of 0.3 dB
• Metamaterial shows significant sensing with a maximum sensitivity of 0.96 THz/RIU

## Graphical abstract

**Figure:**
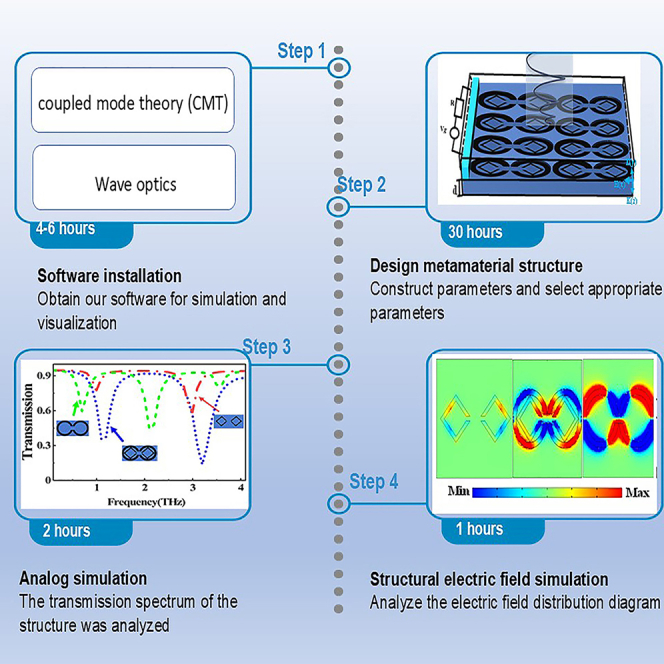

## Introduction

Electromagnetically induced transparency (EIT) refers to a quantum interference phenomenon in atomic systems, which was originally discovered in three-level atomic systems.^1^^,^^2^ The plasma-induced transparency (PIT) results from the synergy of EIT and SPPs. PIT forms through coherent cancellation between broadband bright and narrowband dark modes, akin to symmetric Fano resonance. It finds applications in optical switches, storage, and slow-light devices.^3^^,^^4^^,^^5^ The emergence of two-dimensional material graphene has provided new opportunities for the further development of plasmon-induced transparency sensing technology.^6^^,^^7^ In 2019, Bahadori-Haghighi et al. designed a structure fabricated from bilayer graphene, achieving approximately 95% significant modulation depth (MDA) at a wavelength of 1.55 μm using finite element methods.^8^ In 2022, Cheng et al. not only achieved three-window PIT effects but also attained a MDA of 97.3%.^9^ Zhang et al. proposed a multilayer patterned graphene metamaterial, analyzed using coupled mode theory (CMT), achieving a modulation amplitude of 83.3%.^10^ Tavana et al. introduced graphene structures capable of achieving a modulation amplitude of 91%.^11^ When graphene metamaterial structures are influenced by incident electromagnetic waves, interference cancellation occurs on their surfaces, rendering the graphene metamaterials transparent to the incident electromagnetic waves, thereby achieving the PIT effect. Although all the aforementioned studies have realized the intensity modulation of the transmission window of PIT metamaterials, they all modulate the narrow-band transmission window with complex structure, which limits the practical application of PIT metamaterials to some extent.^12^^,^^13^^,^^14^

This paper presents a wideband tunable metamaterial structure based on the electro-optical properties of graphene metamaterials. The preparation of the structure involves growing a single layer of graphene on a copper foil using chemical vapor deposition (CVD) and transferring it to a silicon-based substrate.^15^ Patterned graphene can be prepared using conventional lithography techniques.^16^ By adjusting the external bias voltage applied to the graphene, the Fermi level of the graphene can be altered, thereby enabling active control over the PIT window.^17^^,^^18^^,^^19^ The simulation results demonstrate that the metamaterial structure exhibits outstanding sensing performance under refractive index variations, with a maximum detection sensitivity of 0.96 THz/refractive index unit (RIU). In comparison to metallic materials with high losses, this graphene metamaterial offers high transmittance, presenting potential applications in optical storage, sensors, and optical switches.

### PIT metamaterial structure

#### Structural parameters of PIT metamaterials

**Figure 1:**
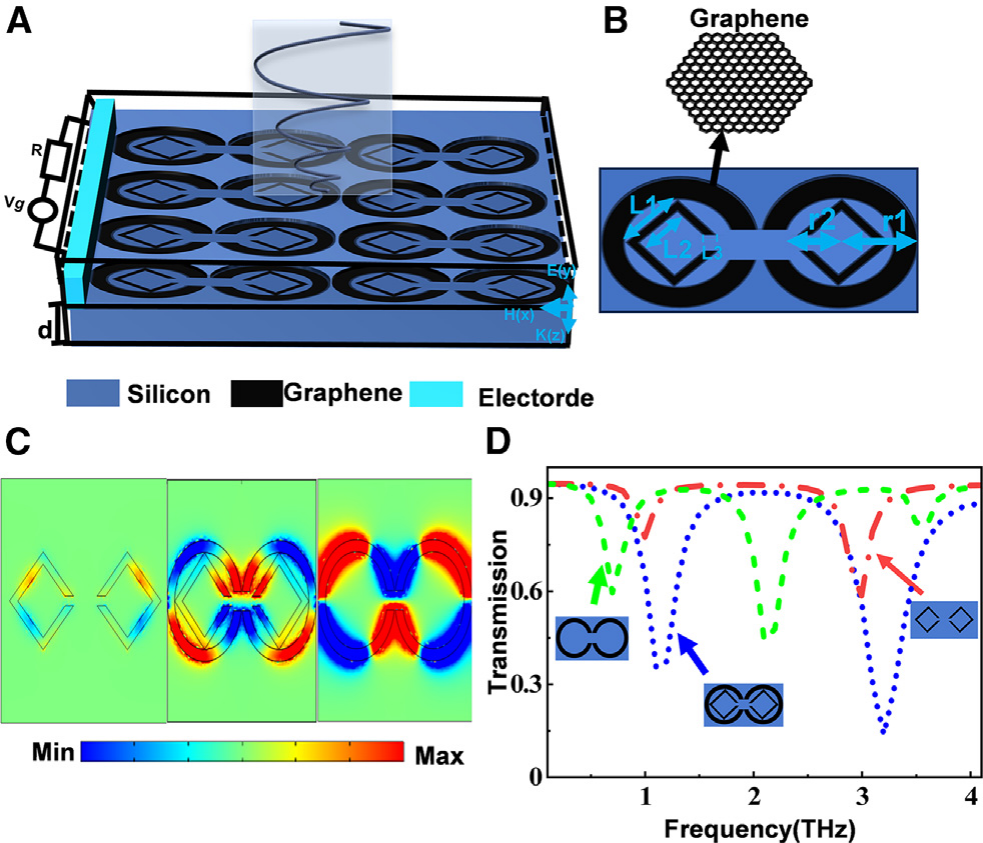
Metamaterial structure and transmission spectrum (A) Three-dimensional schematic diagram of the element structure. (B) A top view of the element structure. (C) Electric field distribution (Ez) in the Z direction for the three structures at f = 2.1 THz. (D) Transmission spectra of different metamaterial structures.

The graphene metamaterial structure is designed in this paper (Figure 1). We further analyzed the field distribution using the finite element method (FEM, COMSOL Multiphysics). In the simulations, periodic boundary conditions were used in the x− and y− directions, Px=Py=10um. The graphene metamaterial layer is deposited on a sapphire substrate with a thickness of d = 100 nm, and we can see a three-dimensional diagram of the component structure composed of graphene and the substrate (Figure 1A). We can also see a top view of the designed metamaterial structure (Figure 1B). The structural parameters are as follows: the distance between the graphene circular rings is $L1=2\sqrt{2}\text{um},L2=\sqrt{4.5}\text{um},L3=0.5\text{um},r1=2.5\text{um},r2=2\text{um}$.

Transmission spectra of different metasurface structures in the terahertz region when the polarization direction of the electric field is along the y axis (Figure 1D). It can be seen that at f = 2.1 THz, only the graphene circular ring structure produces a strong plasma resonance, while at the same frequency, only the graphene square ring structure produces an inductive-capacitive (LC) resonance. When these two structures are combined, they produce the PIT effect, as shown by the blue curve. In order to better understand the origin of the PIT effect, we plotted the electric field distributions of the different hypersurface structures at f = 2.1 THz, where a significant accumulation of electrons is observed on both sides of the nanoring, generating a strong electric field, indicating direct excitation by incident waves, thereby considered to be a bright mode (Figure 1C). When only the graphene square ring exists in the device, the electric field distribution of the graphene square ring is not obvious, which indicates that the graphene square ring cannot be directly coupled with the incident terahertz light, and thus it can be referred to as the dark mode. In this way, the phase cancellation interference between the bright and dark modes gives rise to the PIT effect.

#### Transmission characteristics of the model

To elucidate the mechanism underlying the emergence of the PIT effect, a coupled harmonic oscillator model is used to quantitative describe the near-field coupling between the bright mode and subradiant mode. The proposed destructive interference of graphene-based PIT metamaterial can be described by the following equations:^20^^,^^21^^,^^22^

(Equation 1) $${\ddot{x}}_{1}+\gamma_{1}{\dot{x}}_{1}+{\omega_{0}}^{2}x_{1}+\kappa x_{2}=gE_{0}e^{j\omega t}$$

(Equation 2) $${\ddot{x}}_{2}+\gamma_{2}{\dot{x}}_{2}+{\left(\omega_{0}+\delta \right)}^{2}x_{2}+\kappa x_{1}=0$$

where $x_{1}、x_{2}$ correspond to bright resonator and dark resonator, respectively; $\gamma_{1}、\gamma_{2}$ denote the damping coefficients of the bright and dark resonators, respectively; $\omega_{1}、\omega_{2}$ to denote the intrinsic resonance frequencies of the two resonators, respectively; κ denotes the linear coupling coefficient between these two resonators; g is the geometric coefficient of the coupling strength between the resonator and the incident electric field. By Equations 1 and 2 and $\tilde{x}=\frac{\tilde{x}e}{d}$, We can obtain the magnetization rate of the equipartitioned excitation-induced transparent metamaterials in the class of polarizability $\tilde{x}e$ of the PIT metamaterial unit structure.^23^ d is the thickness of the metamaterial layer. When the equivalent thickness of the PIT metamaterial is small enough, it can be approximated that the incident terahertz waves all pass through the metamaterial layer without reflection, and the transmittance of the PIT metamaterial can be expressed as follows:^24^^,^^25^

(Equation 3) $$\left|\tilde{t}\left(\omega \right)\right|=\left|\frac{{\tilde{t}}_{P-L}\left(\omega \right)}{{\tilde{t}}_{A-M}\left(\omega \right)}\right|$$

where ${\tilde{t}}_{A-M}\left(\omega \right)$ denotes the transmission at the air-substrate interface in the absence of the graphene layer, and this value can be approximated by the Fresnel formula as follows:

(Equation 4) $$\left|{\tilde{t}}_{A-M}\left(\omega \right)\right|=\frac{2}{\left|1+n_{M}\right|}$$

where $n_{M}$ is the refractive index of the substrate. ${\tilde{t}}_{P-L}\left(\omega \right)$ is the Fabry-Perot interferometric transmission coefficient of the metamaterial layer, which can be expressed as follows:

(Equation 5) $${\tilde{t}}_{P-L}\left(\omega \right)=\left|\frac{4n_{P}\;\exp\left(\frac{i\omega dn_{P}}{c}\right)}{\left(n_{P}+1\right)\left(n_{P}+n_{M}\right)-\left(n_{P}-1\right)\left(n_{P}+n_{M}\right)\exp\left(\frac{i2\omega dn_{P}}{c}\right)}\right|$$

where c is the is the speed of light in vacuum and $n_{P}=\sqrt{1+\tilde{x}}$ and is the equivalent refractive index of the PIT metamaterial layer. Since the thickness of the PIT metamaterial layer is much smaller than the incident wavelength, the far-field transmission coefficient can be approximated as the near-field transmission coefficient in the case of *d*→0:

(Equation 6) $$\lim_{d\to 0}\left|\tilde{t}\right|=\left|\frac{c\left(1+n_{P}\right)}{c\left(1+n_{P}\right)-i\omega {\tilde{x}}_{e}}\right|$$

The factors of the Lorentz equation can be fitted by the aforementioned Equations 1, 2, 3, 4, 5, and 6, and the calculation results show that the sensing effect can be realized by changing the refractive index of the substrate, and thus the transmission coefficient, at the incident frequency of high power. Graphene has extremely high electron mobility, which can significantly improve the response speed and sensitivity of the resonator. The optical and electrical properties of graphene can be modulated by external electric fields or chemical doping, providing greater regulatory flexibility than traditional metal or semiconductor materials. In the simulation, the surface conductivity $\sigma_{g}$ of graphene can be expressed as follows:^26^

(Equation 7) $$\sigma_{g}=\sigma_{\mathrm{int}ra}+\sigma_{\mathrm{int}er}=\frac{2e^{2}k_{B}T}{\pi \hbar^{2}}\frac{i}{\omega +\frac{i}{\tau }}\ln\left[2\;\cos\;h\left(\frac{E_{f}}{2k_{B}T}\right)\right]+\frac{e^{2}}{4\hbar_{2}}\left[\frac{1}{2}+\frac{1}{\pi }arctan\left(\frac{\hbar \omega -2E_{f}}{2k_{B}T}\right)-\frac{i}{2\pi }\ln\frac{{\left(\hbar \omega +2E_{f}\right)}^{2}}{{\left(\hbar \omega -2E_{f}\right)}^{2}+4{\left(k_{B}T\right)}^{2}}\right]$$

where $\sigma_{\mathrm{int}ra}$ is the in-band electron photon scattering; $\sigma_{\mathrm{int}er}$ is the interband photon jump; ω denotes the angular frequency of the incident terahertz light; $E_{f}$ is the graphene Fermi energy level; T is the temperature of the built-in work; $k_{B}$ is the Boltzmann constant; e is the meta-charge; $\hbar =\frac{h}{2\pi }$ is the reduced Plank’s constant; $\tau^{-1}=\frac{e{v_{F}}^{2}}{E_{f}\mu_{c}}$ is the relaxation time; $\nu_{F}=10^{6}m/s$ is the Fermi velocity of graphene; $\mu_{C}=3m^{2}/\left(V\cdot s\right)$ is the graphene carrier mobility; at room temperature, in the terahertz band, when the Fermi energy level Ef of graphene is much larger than the photon energy, the in-band jumps can be neglected, therefore, the conductivity of graphene is commonly approximated by the Drude model as follows:^27^

(Equation 8) $$\sigma_{g}=\frac{e^{2}E_{f}}{\pi \hbar^{2}}\frac{i}{\omega +\frac{i}{\tau }}$$

One of the advantages of graphene as a building block for PIT devices is its ability to dynamically control its Fermi energy levels by applying a bias voltage ($V_{g}$):

(Equation 9) $$E_{f}=\hbar \sqrt{\frac{\pi \varepsilon_{0}\varepsilon_{si}V_{g}}{de}}$$

where $\varepsilon_{si}$ and d are the dielectric constant and the distance between graphene and the bias voltage, respectively. $V_{g}$ is the gate voltage. It can be known from the equation of the equipartitioned excitonic wave vector relation for the graphene hypersurface structure that the dynamic regulation of the PIT effect can be realized by changing the graphene Fermi energy level, which is expressed in the relation:^28^^,^^29^

(Equation 10) $$k_{spp}\propto \frac{1}{L}$$

(Equation 11) $$k_{spp}=\frac{\hbar \omega^{2}}{2a_{0}E_{f}c}=\frac{2\pi^{2}\hbar c}{a_{0}E_{f}\lambda^{2}}$$

where $\alpha_{0}=\frac{e^{2}}{\hbar c}$ is a specific constant, λ is the resonant wavelength of the polarized light on the graphene superstructure, and L represents the length of the graphene metamaterial structure. Therefore, the resonance frequency of graphene can be expressed as $f\propto \sqrt{\frac{a_{0}E_{f}}{2\pi^{2}\hbar_{c}L}}$.

## Results and discussion

### Effect of rotation angle

**Figure 2:**
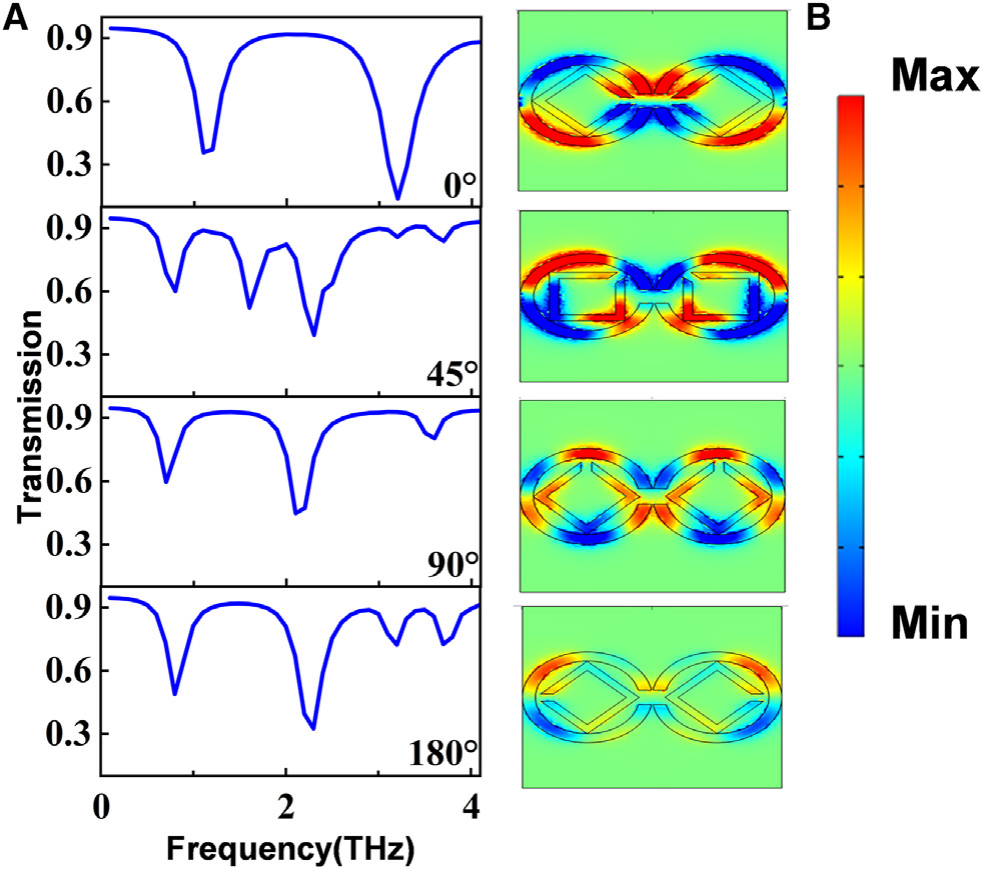
Effect of rotation of angle variation on structural transmission spectrum (A) Transmission spectra corresponding to different rotation angles. (B) Electric field diagrams corresponding to different rotation angles.

The phase cancellation interference of bright and dark modes leads to the generation of the PIT effect. Building upon this, we further analyzed the transmission characteristics of graphene metamaterial structures as the internal graphene square ring rotates.^30^^,^^31^ As the graphene square ring is rotated, the electric field distribution and coupling strength change, resulting in changes in the location and intensity of transmission peaks and valleys. This phenomenon can be visualized and understood by the electric field distribution diagram (Figure 2A). Plot the transmission curves as the position of the internal graphene square ring changes from 0° to 180°, under illumination with y-polarized light. It can be seen that the transmission spectrum is highly sensitive to changes in the rotation angle of the internal graphene square ring, and the electric field distribution corresponding to the structure can also observe this phenomenon (Figure 2B). At θ = 0°, the electric field response is strongest above the opening of the ring, resulting in the maximum interference effect and a single wide transparent window in the PIT spectrum. As the square ring rotates, for example, at θ = 30°, the electric field response weakens and becomes concentrated in the lower half of the opening ring, leading to a reduction in interference effect and a narrower transmission window. A prominent resonant dip appears at 1.6 THz, causing the splitting of the transparent window and a transition from a single PIT window to two windows. When θ = 90°, the electric field response and interference effect decrease, resulting in a wider transmission window. Particularly, at θ = 180°, the electric field response is minimal, resulting in the weakest interference effect, and leading to an increase in the number of PIT windows to three. Consequently, the rotation angle of the graphene square ring alters its coupling strength and phase relationship with the external electromagnetic field. Under different rotation angles, the electric field distribution and current distribution within the internal ring undergo changes, leading to variations in the coupling strength with the external electromagnetic field. These changes directly impact the interference strength between the bright and dark modes, thereby altering the width and position of the transmission window in the PIT effect. The rotation angle variation induces a relative phase change between the internal and external rings. This phase variation affects the phase interference between the bright and dark modes, consequently modifying the position and shape of the PIT transmission window. The rotation angle change causes a redistribution of the electric field within the entire structure, particularly within the internal square ring. Such changes influence the local resonance conditions, consequently altering the manifestation of the PIT effect.

### Dynamic regulation of Fermi energy levels

**Figure 3:**
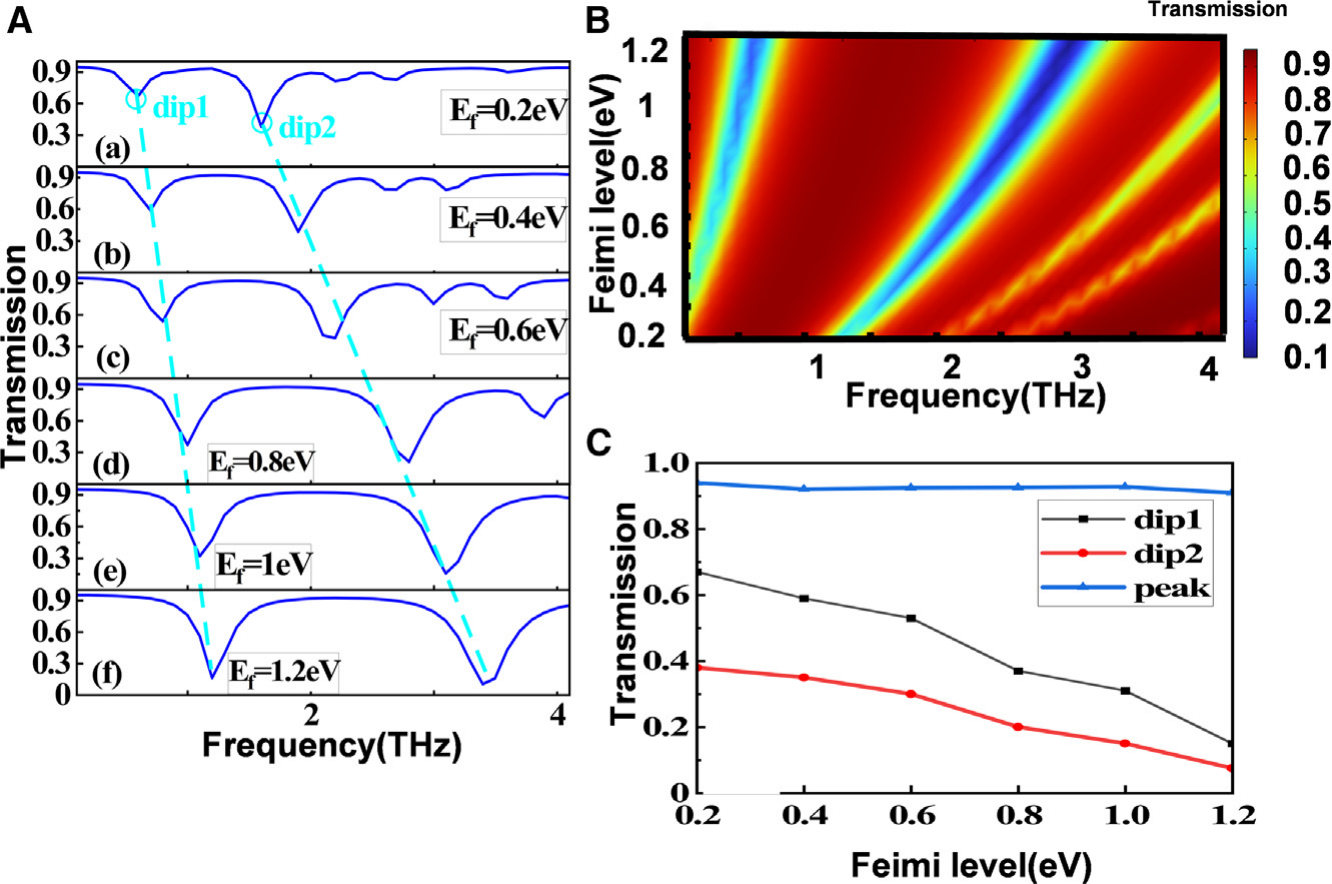
Influence of changing Fermi level on structural transmission spectrum (A) Transmission spectra at different graphene Fermi levels. (B) Three-dimensional demonstration at different Fermi levels. (C) Frequency trends of sharp drops and peak values in the transmission spectrum.

In practical applications, the structure that produces the PIT effect cannot be changed, and therefore the PIT effect cannot be modulated by changing the structural parameters. Our selected graphene metamaterial is characterized by dynamic tuning of conductivity, which can be used to tune the transmission properties of PIT structures by adjusting the Fermi energy levels of graphene.^32^^,^^33^^,^^34^ The increase of Fermi level will cause blue shift in transmission spectrum (Figure 3A). With the increase of Fermi level, the transmittance of the two sharp drop points (dip1 and dip2) decreases, while the peak of transmittance point between dip1 and dip2 (blue curve) increases gently (Figure 3B). The blue shift in the transmission spectra as the graphene fermi level increases is mainly due to the increase in energy required to excite the graphene bands as the fermi level increases, resulting in a blue shift in the resonance peaks.

### Optical switch applications

**Figure 4:**
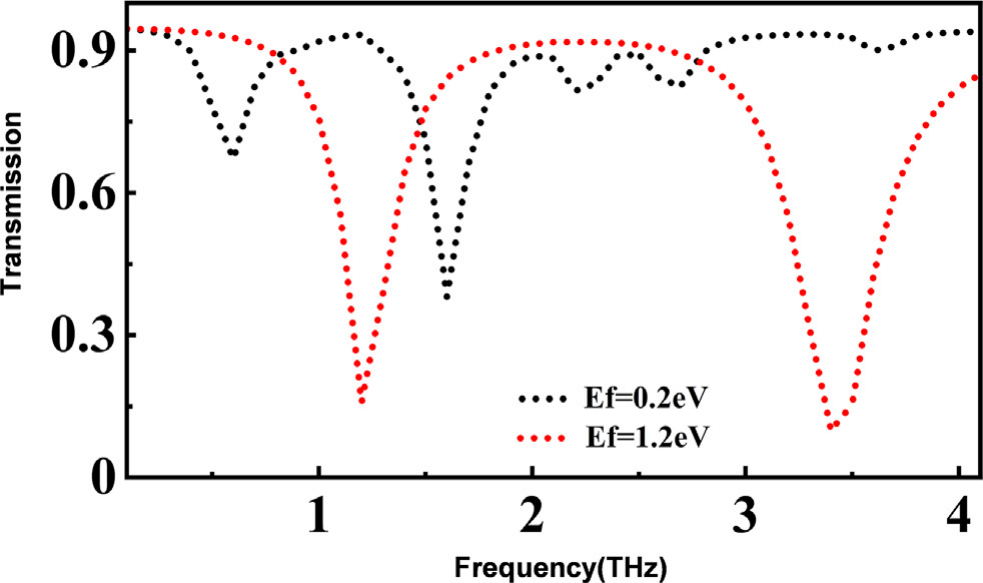
Realization of optical switch

Increasing the Fermi level of graphene can significantly induce a blue shift in the transmission spectrum of the metamaterial structure. This demonstrates the excellent dynamic tunability of the designed structure in terms of transmission spectrum, making it suitable for achieving multi-channel optical switching functions.^21^^,^^35^^,^^36^^,^^37^ We selected transmission spectra with Fermi levels of 0.2 eV and 1.2 eV for investigation. In this study, we set the “on” state threshold of the transmission spectrum to 0.80 and the “off” state threshold to 0.3, corresponding to the binary “1” and “0” in the digital circuit respectively. When the Fermi level is 1.2 eV, the transmittance of the transmission spectrum is lower than 0.3 in the range of 3.3–3.6 THz, while when the Fermi level is 0.2 eV, the transmittance of the transmission spectrum in the above band is higher than 0.8 (Figure 4). Therefore, any frequency within this terahertz band can be prepared into a frequency optical switch. When E_f_ = 1.2 eV, the optical switch is everywhere in the “off” state at 1.2 THz and 3.4 THz, and when E_f_ = 0.2eV, the transmission peak of the transmission spectrum corresponds to these two terahertz frequencies, so the optical switch can be set to the “on” state. In this way, we have achieved a two-frequency optical switch.

To better evaluate the performance metrics of the optical switch, we typically introduce several interacting parameters including MDA, insertion loss (IL), and ER.^10^^,^^38^^,^^39^^,^^40^^,^^41^^,^^42^ They are defined as follows:

(Equation 12) $$MDA=\frac{\left|T_{on}-T_{off}\right|}{T_{on}}\times 100\%$$

(Equation 13) $$IL=-10\;\log_{10}T_{on}\left(dB\right)$$

(Equation 14) $$ER=10\;\log_{10}\frac{T_{on}}{T_{off}}\left(dB\right)$$

In this context, T_on_ and T_off_ represent the transmission amplitudes in the “on” and “off” states, respectively, at a certain frequency during the entire modulation cycle. The lower the value of IL that can be achieved while keeping the values of MDA and ER as high as possible for the designed structure, the better the performance of the optical switch is indicated.

We can calculate that at 3.4 THz, the maximum values of MDA and ER, along with the minimum value of IL, are 91%, 10.94 dB, and 0.3 dB, respectively, with a bandwidth of 0.3 THz. Additionally, the designed structure in this study also functions effectively in certain terahertz bands for optical switching.

We also give a comprehensive comparison of the properties of the graphene metamaterial structures designed in this paper with other metamaterial structures (Table 1).When MDA reaches 91%, IL reaches 0.3 dB at the lowest, which is superior in performance compared with literature.^40^^,^^41^^,^^43^^,^^44^ For the literature, although high MDA and ER values can be achieved, IL is not within the range of 0.3 dB.^45^ The structure we designed can ensure that MDA > 90%, ER > 10 dB, and insertion loss (LD) < 0.3 dB. It shows intuitively that the structure we designed can ensure MDA > 90%, ER > 10 dB, and IL < 0.3 dB; on the other hand, the structure proposed in this paper is much simpler compared with other structures, realizing four inductive transparent windows in a single-layer graphene structure, which makes the asynchronous switch realization possible. In summary, our proposed superstructure has obvious advantages over other structures in terms of overall performance.

**Table 1:** Comparison of the proposed structured light opening with other structured light openings

Material structure	Optical effect	Modulation mode	MDA (%)	IL (dB)	ER (dB)	References
Multilayer graphene metamaterials	Triple-window PIT	Multiple-frequency	87.8%	0.31	9.15	Liu et al.^43^
Bilayer graphene metamaterials	Single- window PIT	Triple-frequency	86.1%	8.1	8.58	Li et al.^44^
Multilayer graphene metamaterials	Dual-window PIT	Single-frequency	83.3%	0.33	7.77	Xiong et al.^46^
Bilayer graphene metamaterials	Single-window PIT	Triple- frequency	77.7%	16.3	12.5	Zhang et al.^42^
Multilayer graphene metamaterials	Dual-window PIT	Multiple-frequency	96.3%	0.33	18.89	Sarker et al.^45^
Single-layerpatterned graphene	Single-window PIT	Quad- frequency	91%	0.3	10.94	This work

### Sensing properties of structures

The generation of the PIT effect is often accompanied by a strong energy localization effect, which makes it very sensitive to changes in the refractive index of the surrounding environment and has the potential to become a high-performance sensor.^47^^,^^48^^,^^49^ Sensitivity (S), quality factor (Q), and figure of merit (FOM) are performance indicators of a sensor, the specific expression is as follows:^50^^,^^51^^,^^52^

(Equation 15) $$S=\frac{\Delta f}{\Delta n}$$

(Equation 16) $$FOM=\frac{S}{FWHM}$$

where Δf refers to the resonance frequency shift of the superstructure, Δn refers to the refractive index change of the substrate, and FWHM is the full width at half of the transparency tilt angle. The overall sensing performance of the sensor is mainly measured by the FOM value; the larger the FOM value, the larger the sensitivity S and Q of the sensor have reached the ideal index.^53^

**Figure 5:**
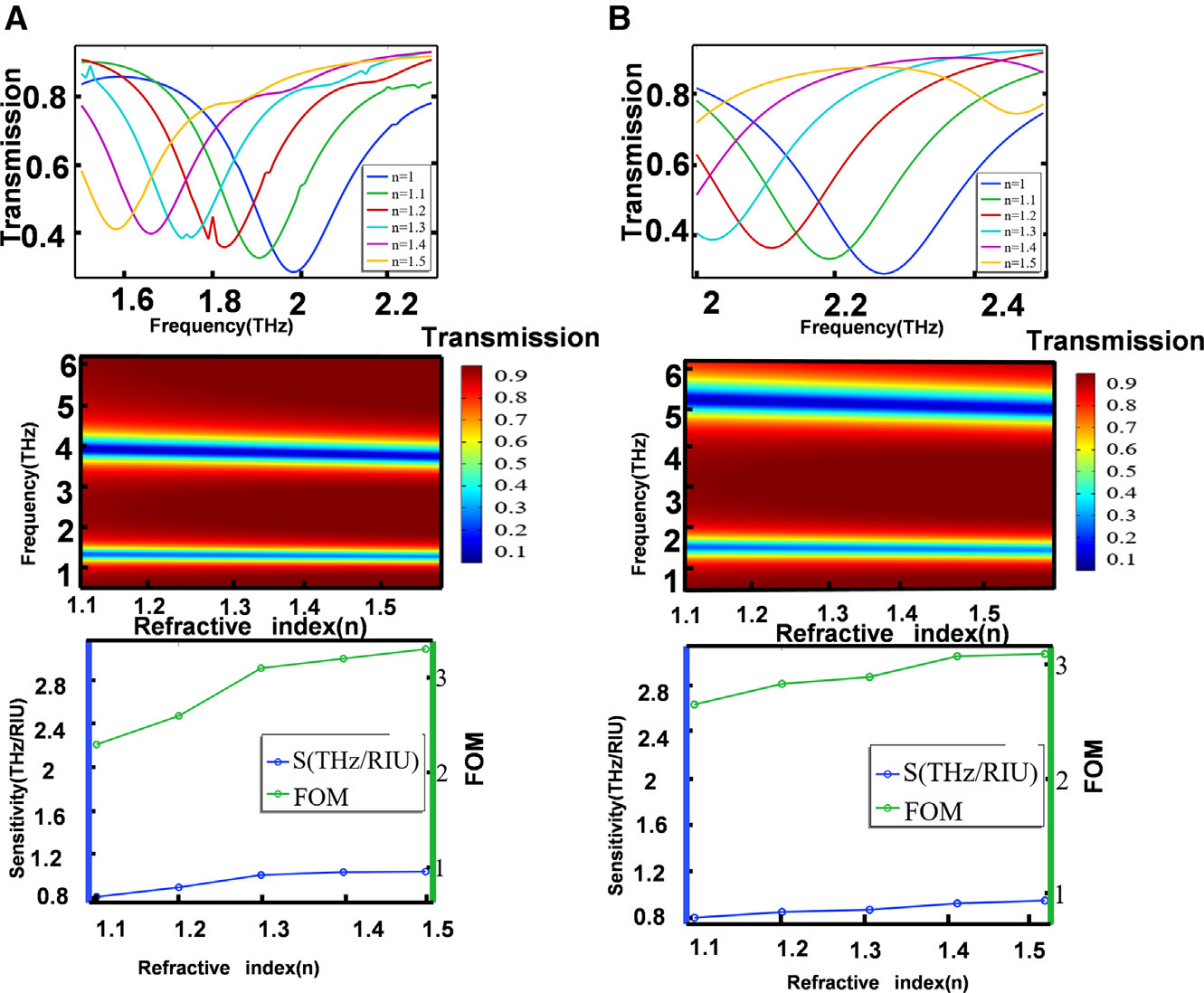
Determination of superstructure sensitivity (A) Determination of sensitivity under x polarized light. (B) Determination of sensitivity under y polarized light.

During the simulation, the substrate material is set to be lossless. When the ambient refractive index n increases from 1.00 to 1.50, the transmission spectrum of the superstructure under y-polarization irradiation shows an obvious redshift, and the sensitivity S and FOM values of the superstructure increase with the increase of the ambient refractive index. The maximum detection sensitivity that the sensor can achieve in the y polarization direction is S_1_ = 0.92 THz/RIU, FOM_1_ = 3.3 (Figure 5A). Similarly, with the increase of refractive index, the transmission spectrum of the superstructure under x polarization irradiation also shows an obvious redshift phenomenon, and the sensitivity S and FOM values of the superstructure also increase. The maximum detection sensitivity that can be achieved in the x polarization direction is S_2_ = 0.96 THz/RIU, and FOM_2_ = 3.2 (Figure 5B). This shows that the superstructure has good sensing performance to the change of refractive index.

we found that as the refractive index varies from 1.00 to 1.50, the maximum sensitivity of our designed terahertz metamaterial structure reaches 0.96 THz/RIU, whereas the sensitivities of the structures listed in the table are all below 0.5 THz/RIU (Table 2). The sensitivity of the structure we designed exceeds that of the team in the table, and as a result, the sensor has excellent overall sensing performance (Table 2).^54^ In the design of refractive index sensors, we have learned that we can use feature mapping methods to optimize data mapping through deep learning, enhance sensor sensitivity, and overcome high-dimensional data processing problems. This provides us with ideas for further research.^55^

**Table 2:** Comparison of the sensitivity of the proposed graphene hypersurface with other previously reported structures

Nanostructure type	Refractive index range	Sensitivity (THz/RIU)	FOM	References
Three SRR	(1.5–2.0)	0.28	6.9	Zhu et al.^56^
Triadius and eSRR	(1.0–1.6)	0.379	63.83	Zhong et al.^57^
Carbon nanotube	(1.1–1.4)	0.74	1.85	Li et al.^58^
Single-layer graphene	(1–1.08)	0.91	15.88	Meng et al.^59^
patterned graphene	(1.0–1.5)	0.96	3.3	This work

### Conclusion

This paper proposes a graphene-based metamaterial structure composed of graphene ring pairs and graphene square ring pairs, exhibiting PIT effect. By adjusting the Fermi level of graphene, the transmission characteristics of the PIT structure can be modulated to achieve a wideband tunable PIT effect. Simulation and theoretical calculation show that the structure can realize the two-frequency optical switch in the range of 0–4.1 THz, the maximum value of MDA and ER are 91% and 10.94 dB, respectively, and the minimum value of IL is 0.3 dB, which has excellent performance. In the 3.3–3.6 THz range, the structure can achieve broadband adjustable transmission amplitude, expanding its potential application range. By varying the substrate refractive index, the sensing characteristics of the metamaterial structure are revealed, with the highest sensitivity of the metamaterial reaching 0.96 THz/RIU, surpassing previous metamaterial structures. Furthermore, this structure features a simple design and convenient modulation methods, holding potential application value in terahertz switches, terahertz filters, and terahertz modulators. The actual manufacturing process requires high precision, and in the next phase of work, we will explore simpler manufacturing methods to reduce costs and improve repeatability.

### Limitations of the study

There are many limitations and difficulties in calculating graphene pattern layers using FEM. These challenges are mainly due to the functional limitations of the simulation software itself, the limitations of computational resources, the complexity of graphene material properties, and the difficulties of experimental preparation and verification.

## Resource availability

### Lead contact

Requests for further information and resources should be directed to and will be fulfilled by the lead contact, Liuli Qin (qinliuli@126.com).

### Materials availability

This study did not generate new materials.

### Data and code availability

• All data generated or analyzed during this study are included in the manuscript.
• Any additional information required to reanalyze the data reported in this paper is available from the lead contact upon request.

## Acknowledgments

The authors would like to thank 10.13039/501100001809National Natural Science Foundation of China (grant no. 51965007); 10.13039/100012547Guangxi Natural Science Foundation (2023GXNSFAA026015); Key Laboratory of Advanced Electrode Materials for Novel Solar Cells for Petroleum and Chemical Industry of China, School of Chemistry and Life Sciences, Suzhou University of Science and Technology, Suzhou City, Jiangsu Province 215009 (P.R. China); and The Animal Care and Protection Committee of Guangxi Normal University. Funding was provided by Key Laboratory of Advanced Electrode Materials for Novel Solar Cells for Petroleum and Chemical industry of China, School of Chemistry and Life Sciences, 10.13039/501100004716Suzhou University of Science and Technology (grant no. 2024A103).

## Author contributions

J.Z. conducted experiments and wrote the manuscript under the supervision of X.C. and L.Q. contributed to picture designs. All authors contributed to the draft and prepared the final version.

## Declaration of interests

The authors declare no competing interests.

## STAR★Methods

### Key resources table

**Table:** 

REAGENT or RESOURCE	SOURCE	IDENTIFIER
**Software and algorithms**		

Windows 10	Microsoft	https://www.microsoft.com/en-us/software-download/windows10

**Other**		

CPU laptop	Lenovo	Intel Core i5-12600kf

### Method details

#### Installation of system resources and applications

We deploy the protocol on Windows 10 Pro desktop computers. The desktop computer uses an Intel Core i5-12600kf CPU.The simulation environment is built and the finite element method is used to calculate.

#### Setting of metamaterial structure

Periodic boundary conditions are set in the x and y directions, and the light source enters from the z direction.

#### Structural transmission spectrum analysis

The finite element method (FEM) enables the simulation and analysis of interactions between various physical fields, including electromagnetic, sound and thermal fields. In optics, electronic device design, material science and other fields. Structural transmission spectrum analysis is one of the important means to study the propagation and interaction of light in materials. Through the analysis of transmission spectrum, we can deeply understand the optical properties of materials, optical resonance phenomenon and the mechanism of interaction between light and matter.

#### Electric field response analysis

The finite element method is used to solve the model, and the potential value and electric field distribution of each point are obtained by numerical calculation. According to the distribution of electric field and electric field lines, the structure of the metamaterial is optimized. By adjusting material geometry, material parameters or loading conditions, the uneven distribution of electric field can be improved and the performance of metamaterials can be improved.

#### Expected outcomes

Transmission spectra of different metasurface structures in the terahertz region when the polarization direction of the electric field is along the Y-axis (Figure 1D). It can be seen that at f = 2.1 THz, only the graphene circular ring structure produces a strong plasma resonance, while at the same frequency, only the graphene square ring structure produces an LC resonance. When these two structures are combined, they produce the PIT effect, as shown by the blue curve. In order to better understand the origin of the PIT effect, we plotted the electric field distributions of the different hypersurface structures at f = 2.1 THz, where a significant accumulation of electrons is observed on both sides of the nanoring, generating a strong electric field, indicating direct excitation by incident waves, thereby considered to be a bright mode (Figure 1C). When only the graphene square ring exists in the device, the electric field distribution of the graphene square ring is not obvious, which indicates that the graphene square ring cannot be directly coupled with the incident terahertz light, and thus it can be referred to as the dark mode. In this way, the phase cancellation interference between the bright and dark modes gives rise to the PIT.
